# Application of Advanced Imaging to the Study of Virus–Host Interactions

**DOI:** 10.3390/v13101958

**Published:** 2021-09-29

**Authors:** Cristina Risco

**Affiliations:** Cell Structure Laboratory, Centro Nacional de Biotecnología (CNB-CSIC), Campus UAM, Cantoblanco, 28049 Madrid, Spain; crisco@cnb.csic.es

## 1. Introduction

Recent advances in light and electron microscopy are uncovering viral lifecycle events with a level of detail never before seen. Advanced imaging is also helping to unveil new targets for antiviral intervention and to facilitate the use of viruses as tools in biotechnology and to cure disease.

This Special Issue of *Viruses* contains 13 papers, including 8 reviews, 4 articles and 1 brief report, presented by scientists working in Portugal, South Africa, Australia, the Czech Republic, France, the United Kingdom, Spain, Germany, The Netherlands, and the USA. Together, they devise a variety of imaging techniques to study virus–host interactions, such as single-molecule super-resolution imaging, quantitative live cell microscopy, super-resolution microscopy, volume scanning electron microscopy (SEM), transmission electron microscopy (TEM), electron tomography, imaging techniques for whole organs, labeling methods for proteins and nucleic acids, including immunogold, labeling with nucleotide analogs, in situ hybridization and EM autoradiography, and strategies to fluorescently label viral and cellular components.

**Figure 1:**
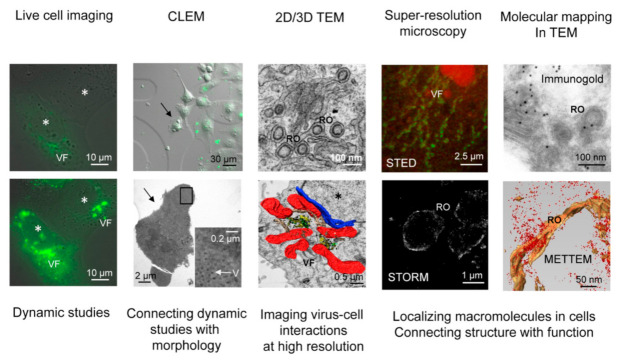
Visual introduction to imaging virus–host interactions. Images were obtained by live-cell microscopy, correlative light and electron microscopy (CLEM, arrows point to the same cell as visualized by light and electron microscopy), two- and three-dimensional transmission electron microscopy (TEM), STED (Stimulated Emission Depletion), STORM (Stochastic Optical Reconstruction Microscopy), and techniques for molecular mapping in TEM, such as immunogold and metal-tagging TEM (METTEM). The authors of the images are Dr. Isabel Fernández de Castro and Dr. Raquel Tenorio (CSL, CNB-CSIC), Dr. Orestis Faklaris (MRI-Biocampus, Montpellier), Dr. Laura Sanz-Sánchez (Ecogen) and Dr. Cristina Risco (CSL, CNB-CSIC). VF, viral factory; V, viral particle; RO, viral replication organelle; asterisk, cell nucleus.

Some of the methods used to study virus–host interactions are introduced in Figure 1. The figure contains images from live-cell microscopy, correlative light and electron microscopy (CLEM), two- and three-dimensional TEM, super-resolution microscopy methods such as STED (Stimulated Emission Depletion) and STORM (Stochastic Optical Reconstruction Microscopy), and techniques for molecular mapping in TEM, such as immunogold and metal-tagging TEM (METTEM).

This Special Issue shows how imaging methods are crucial in studying infections from plant viruses, the African Swine Fever Virus, the human polyomavirus, influenza virus, Ebola virus, the respiratory syncytial virus, adeno-associated viruses, coronaviruses, nodaviruses, enteroviruses, adenoviruses, and the human immunodeficiency virus. Highlights of the works are summarized in the next three sections dedicated to reviews and articles about light microscopy, electron microscopy, and both.

## 2. Light Microscopy

Peter et al., [1] present a live cell microscopy study of the subnuclear replication centers assembled by the murine polyomavirus. With high throughput widefield microscopy, spinning disc confocal microscopy, and spatiotemporal analysis of live cell microscopy, in this article the authors study viral replication in live cells and inside subnuclear domains, characterizing the temporal dynamics of viral replication centers (VRC) formation and expansion over the course of infection. Their results show the dynamic nature of VRCs and how host proteins incorporate at different stages, as well as how the multiplicity of infection affects VRC dynamics.

The article by Yuan et al., [2] is a single-molecule super-resolution imaging study of CD4 redistribution in T-cell plasma membrane upon HIV-1 binding. Single-molecule localization microscopy (SMLM) techniques such as STORM allow individual cell-surface proteins to be mapped on intact cells. The article shows the precise way in which HIV-1 receptors redistribute to sites of virus binding. The quantitative approach provides a robust methodology for characterizing the nanoscale organization of plasma membrane receptors in general with the potential to link spatial organization to function.

The review by Mukherjee et al. [3] summarizes strategies to study the early phase of HIV-1 infection by fluorescence microscopy. Quantitative microscopy and multi-color imaging studies have brought to light new data on the dynamics of the early stages of infection, from entry to integration in the nucleus, as well as the role of host cell factors participating in infection. The review shows a combination of labeling strategies that have been key in these studies and a description of how single virus tracking has helped to identify the small fraction of viral particles that triggers productive infection.

In his review, Saffarian [4] highlights the contribution of advanced light microscopy techniques to the characterization of human immunodeficiency virus (HIV) assembly, release and maturation. This review places special emphasis on live imaging approaches such as Total Internal Reflection Fluorescence (TIRF), high-resolution light microscopy techniques including PALM and STORM, and single molecule measurements, including Fluorescence Resonance Energy Transfer (FRET). Other approaches, such as Fluctuation spectroscopy techniques and their capacity to utilize the dynamic nature of biological processes, Fluorescence Recovery after Photobleaching (FRAP), and Fluorescence Resonance Energy Transfer (FRET), are also discussed.

The review by Touzier et al. [5] presents new advances in the characterization of influenza virus infection thanks to new optical imaging tools, such as quantitative live-cell and super-resolution microscopy. The authors show how these methodologies are helping to perform a spatio-temporal analysis of influenza virus replication and to identify new opportunities for developing antiviral strategies. For future studies, they describe how advanced fluorescence microscopy techniques will help to understand influenza virus structures and associated host-cell components at the nanoscale, resolve individual viruses among large populations of viruses, and observe dynamic processes in real time.

Maolin [6] presents a review on single-molecule Förster resonance energy transfer (smFRET) imaging of virus spike–host interactions and highlights how this technique connects structures with the function of spikes of SARS-CoV- 2, HIV-1, influenza, and Ebola viruses. The review focuses on how smFRET imaging has advanced our understanding of virus spikes’ dynamic nature. Combining smFRET, super-resolution, and cryoEM/cryoET will make it possible to dissect every single step of viral membrane fusion to develop therapeutic interventions because virus spike proteins are primary targets for vaccines and anti-viral treatments.

The review by Junod et al. [7] describes high-speed single-point edge-excitation sub-diffraction (SPEED) microscopy for tracking the nuclear import of adeno-associated viruses (AAV) through the nuclear pore complexes (NPCs) in live human cells. Understanding the detailed nuclear import kinetics will help to use AAV capsids as a nuclear delivery instrument, as well as a target for drug development. The review contains detailed descriptions of methods, equipment, sample preparation protocols, and data analysis.

In their brief report, Frétaud et al. [8] combine tissue clearing and 3D deep imaging of the entire mouse lung to visualize Respiratory Syncytial Virus (RSV) infection at a cellular resolution. Deep tissue imaging using light-sheet or 2-photon microscopy allowed them to visualize the distribution of the RSV-infected cells in their tissue environment. Using these techniques, biopsies, organoids, or organisms can be processed to study complex biological pathways in three dimensions and to gain insight into host–pathogen interactions in different physiological conditions.

## 3. Electron Microscopy

Wolff and Bárcena [9] present a review that is a chronological account of the progress of electron microscopy techniques that provide structural and functional information at a wide range of scales. The membranous viral replication organelles (ROs), where RNA viruses synthesize their genome, are shown with a variety of EM techniques, such as FIB-milled cryo-lamellae, a volume SEM method, and in situ cryotomography, together with immunogold and autoradiography molecular mapping techniques. EM studies with coronaviruses, enteroviruses, nodaviruses and flaviviruses are showing the macromolecular complexity of viral ROs.

The review by Baena et al. [10] is a practical introduction to focused ion beam scanning electron microscopy (FIB-SEM), one of the volume electron microscopy approaches to study cellular architecture and virus–host interactions. The authors discuss practical aspects of a room-temperature FIB-SEM experiment showing an in vitro study of SARS-CoV-2 infection.

## 4. Light and Electron Microscopy

The article by Aicher et al. [11] studies African swine fever virus (ASFV) replication sites using bioconjugation with click IT chemistry in combination with stimulated emission depletion (STED) microscopy, EM and ET. Early stages of infection were analyzed in cells processed by high-pressure freezing and freeze-substitution, and immunogold on thawed cryosections. The study provides new data on ASFV replication factory biogenesis and evolution.

Sánchez-Pina et al. [12] present a review about imaging techniques to study plant virus replication and plant-to-plant vertical transmission. The authors describe macroscopic techniques such as tissue-printing hybridization and microscopy techniques including light microscopy, confocal laser scanning microscopy, and scanning and transmission electron microscopies and their contributions to plant virology.

The article by Condezo and San Martín [13] describes methods for labelling viral nucleic acids in the cell and show their experience with adenoviruses. They focus on strategies based on the use of nucleotide analogs such as bromodeoxyuridine (BrdU) or bromouridine (BrU). In adenovirus infections, BrdU has been used to localize newly synthesized viral genomes in the nucleus, where it is key to distinguish between host and viral DNA. The article describes methodological variations of BrdU labeling to localize adenovirus genomes in fluorescence and electron microscopy, with a practical discussion about problems and solutions.

## 5. Concluding Remarks

This Special Issue contains a collection of articles and reviews that represent an update on the most advanced methodologies for imaging virus–host interactions. Imaging in virology and cell biology is facing important challenges for the future, such as the development of new tools for image processing and more robust methods to label macromolecules in 3D volumes. Together with the methodological developments in imaging, advances in sample preparation have been key for the advances in the field. In the articles and reviews of the Special Issue, readers can find relevant information about sample preparation for microscopy. We hope that people interested in cell–pathogen interactions or curious about cell architecture will find some clues about how imaging is helping us to learn more about viruses in their hosts.
